# Use of Impella RP Flex in Post-Heart Transplant Patients with RV Primary Graft Dysfunction

**DOI:** 10.3390/biomedicines13061335

**Published:** 2025-05-29

**Authors:** Ioana Dumitru, Jonathan DeWolf, Maria Sevillano, Leeandra Schnell, Hiram Bezerra, Debbie Rinde-Hoffman

**Affiliations:** 1 TGMG Cardiology, Tampa General Hospital, Tampa, FL 33606, USA; mariaesevillano@usftgp.org (M.S.); leeandraschnell@usftgp.org (L.S.); drhoffman@tgmg.org (D.R.-H.); 2 Alabama College of Osteopathic Medicine, Dothan, AL 36303, USA; dewolfj7392@acom.edu; 3 Interventional Cardiology, University of South Florida, Tampa, FL 33606, USA; hbezerra@usf.edu

**Keywords:** heart transplantation, heart-assist devices, right ventricular dysfunction, primary graft dysfunction

## Abstract

**Background**: Right ventricular primary graft dysfunction (RV-PGD) is a rare but serious complication following heart transplantation, associated with a high morbidity and mortality. Temporary mechanical circulatory support is indicated when patients fail to respond to pharmacological therapy. This study aimed to evaluate the outcomes of patients with RV-PGD who received RV mechanical support with the Impella RP Flex device at our institution. **Methods**: Medical records of patients with RV-PGD supported by the Impella RP Flex device between December 2022 and March 2024 were reviewed retrospectively to assess survival, procedural complications, duration of support, and end organ dysfunction. **Results**: Of the 20 patients reviewed, 5 met the inclusion criteria. All five patients demonstrated recovery of RV function after a mean support duration of 8.6 ± 3.05 days. One pump showed transient evidence of biologic material ingestion during a weaning trial. No cases of tricuspid valve injury were observed. The most common complications were hemolysis, bleeding, and acute kidney dysfunction, with all patients requiring hemodialysis. **Conclusions**: Impella RP Flex support is safe and effective for managing primary and isolated RV-PGD without the need for additional blood oxygenation. However, bleeding complications requiring intervention remain a significant concern, and further evaluation of renal recovery is warranted.

## 1. Introduction

Right ventricular primary graft dysfunction (RV-PGD) is a rare but serious complication following heart transplantation, characterized by isolated RV failure in the immediate postoperative period [1,2]. Depending on the diagnostic criteria, the incidence of RV-PGD is generally lower relative to left-sided or biventricular PGD [3,4,5,6]; however, RV-PGD remains clinically significant due to its high morbidity and mortality [3,7]. The management of isolated right-sided failure poses unique hemodynamic challenges, and treatment options remain limited, particularly given the lack of targeted pharmacological therapies or robust randomized data to guide device-based support strategies [2,7]. Applying the 2014 International Society for Heart and Lung Transplantation (ISHLT) consensus definition for RV-PGD, a recent meta-analysis of studies published between 2014 and 2020 reported an incidence of RV-PGD at 1.6% among 2912 heart transplant recipients in 14 separate studies, with a one-year mortality rate of 35% based on data from 450 patients in two of the studies, underscoring the substantial impact of this complication despite its low frequency [3].

Currently, there is no established severity grading scale for RV-PGD diagnosis, which hinders efforts at standardizing management or prognostication [1,2,3]. The current ISHLT definition [2] relies on either hemodynamic evidence of isolated RV dysfunction or the requirement for mechanical circulatory support (MCS) with an RV assist device (RVAD) within 24 h post-transplantation [1,2,8]. Hemodynamic parameters include a right atrial pressure (RAP) greater than 15 mmHg, pulmonary capillary wedge pressure (PCWP) less than 15 mmHg, cardiac index (CI) less than 2.0 L/min/m^2^, and transpulmonary pressure gradient (TPG) less than 15 mmHg and/or pulmonary systolic arterial pressure less than 50 mmHg [1,2,7]. Unlike PGD involving the left ventricle, the lack of severity grading in RV-PGD limits the ability to risk-stratify patients and compare outcomes across multiple cohorts.

The pathophysiology of PGD is multifactorial, resulting from cumulative insults to the donor heart during procurement, preservation, implantation, and reperfusion [1,2]. Four distinct injury phases have been described: brainstem death, cold ischemia during storage, warm ischemia during implantation, and ischemia–reperfusion injury post-anastomosis [1,2,7]. Donor brain death can trigger a surge of catecholamines, leading to myocardial injury and an elevated afterload in both ventricles. Inflammatory and metabolic derangements, including calcium overload and endocrine dysregulation, further impair myocardial reserve [1,2]. Cold ischemia reduces cellular metabolism and also predisposes the donor heart to mitochondrial dysfunction and oxidative injury. Reperfusion itself triggers calcium overload and oxidative stress, activating mitochondrial permeability transition pores and contributing to cardiomyocyte death [1,2]. Animal studies support these mechanisms, and it has been demonstrated that both left and RV diastolic function decline after prolonged cold ischemia, with measurable impairment in RV compliance after just two hours of cold storage at 4 °C [9].

A wide range of donor, recipient, and procedural factors contribute to the risk of PGD [1,2,3,7,8,10]. Key donor characteristics linked to increased risk include advanced age, donor/recipient transplant mismatches in heart size and gender (female-to-male), severe pre-transplant conditions such as dependence on intravenous inotropic support, mechanical circulatory support, or mechanical ventilation, pre-existent pulmonary hypertension, and prolonged surgical durations due to blood transfusions [1,2,3,7,10]. The RADIAL risk score, which incorporates donor and recipient factors, such as a donor age over 30 years, recipient age of 60, diabetes, elevated right atrial pressure, inotrope dependence, and ischemic time over 240 minutes (min), has been proposed to predict PGD, although it may not reliably identify patients at risk of severe disease [2,3,7]. Importantly, female recipient sex and elevated bilirubin have also been independently associated with severe RV-PGD [2,3,7,10]. The growing use of donation after circulatory death (DCD) donors introduces further complexity, as these hearts may experience longer warm ischemia and increased pulmonary vasoconstriction, potentially worsening RV performance, though outcomes remain comparable in carefully selected DCD vs. donation after brain death transplants [1,7].

Clinically, RV-PGD is often marked by hypotension, elevated central venous pressure, and low cardiac output [7]. Myocardial edema, worsened by inotrope-induced calcium overload, may also contribute to early RV failure and delayed recovery [7]. Recovery typically occurs within two weeks, but the time course is variable and can be prolonged in cases with multi-organ dysfunction.

The initial treatment for RV-PGD focuses on medical optimization, including preload reduction, inotropic support, and pulmonary vasodilation using agents such as milrinone, epinephrine, and inhaled nitric oxide [1,2,7]. The mechanical removal of excess fluid is further recommended for RV unloading and can be achieved through intravenous diuretics or dialysis. However, many patients fail to respond to pharmacological therapy alone. For those with persistent hemodynamic compromise, early initiation of temporary mechanical circulatory support is indicated to prevent end organ dysfunction, refractory hypotension, and progressive RV deterioration [2,7]. In the case of isolated RV failure, options include the Impella RP Flex (Abiomed, Danvers, MA, USA), the ProtekDuo (LivaNova, London, UK), and the CentriMag (Abbott Cardiovascular, Plymouth, MN, USA), each with unique insertion routes and flow profiles [7]. In cases of concurrent LV dysfunction or the need for oxygenation, RV-PGD can also be treated with venous arterial ECMO (VA-ECMO) [7]. If recovery does not occur, patients may be re-evaluated for a redo orthotopic heart transplant [1].

To date, few studies have reported on the safety and efficacy of RVADs for RV-PGD, highlighting the need for further research to determine the effectiveness of the available mechanical support devices [7]. This study discusses the cases of five patients with RV-PGD that were treated with the Impella RP Flex as a temporary MCS (tMCS) device. The Impella RP Flex is a minimally invasive, percutaneous, single vascular-access pump designed for right heart support and is one of only two percutaneous RVADs approved by the U.S. Food and Drug Administration (FDA) for the treatment of RV failure (RVF). A recent prospective study of 60 patients treated with the Impella RP for RVF, including seven heart transplant recipients, reported that 73.3% of patients met the primary survival endpoint with minimal complications, such as bleeding [11]. The objective of this study was to retrospectively analyze the outcomes of patients with RV-PGD who received mechanical circulatory support with the Impella RP Flex at our institution.

## 2. Materials and Methods

### 2.1. Study Population

For this retrospective study, we analyzed the medical records of patients who underwent heart transplantation and subsequently experienced RV-PGD. These patients were treated at Tampa General Hospital between December 2022 and March 2024, and received tMCS with an Impella RP Flex device within 48 hours (h) of the transplant procedure. Tampa General Hospital is a quaternary academic institution with a high-volume heart transplant program, performing more than 50 heart transplants annually, along with other solid organ transplant programs.

### 2.2. Study Design

The study design is observational, retrospective, and descriptive. The retrospective design of this study eliminates the need for informed consent.

Anonymized patient records were reviewed to collect pre-Impella RP Flex implant patient demographic data, heart failure etiology, inotrope use, laboratory values, and tMCS device use. Donor organ characteristics, clinical parameters during Impella RP flex support, survival, complications, days of support (DOS), and end organ dysfunction or recovery were also assessed. Given the limited sample size, formal statistical analyses were not performed. Instead, the findings are presented to illustrate trends observed within this small patient cohort.

## 3. Results

### 3.1. Patient Characteristics

During the retrospective study period, a total of 20 patients were treated with the Impella RP Flex device, of whom 5 received support specifically for post-heart transplant RV-PGD. The patients were male, with an average age of 54.4 [range 40–68] years old. The heart failure etiology among these patients included three with non-ischemic dilated cardiomyopathy, one with ischemic cardiomyopathy, and one with transplant coronary artery disease requiring re-transplantation (Table 1). Transplant procedures varied: three patients underwent heart-only transplants (including one re-transplantation), one patient received a combined heart and liver transplant, and one patient received a combined heart and kidney transplant. Pulmonary hypertension was present in three of the five patients, and two patients received tMCS (intra-ortic balloon pump [IABP] and biventricular Impella) prior to heart transplantation. Three patients received intravenous heparin and inotrope therapy with milrinone (0.25–0.5 mcg/kg). Serum creatinine levels ranged from 0.9–5 mg/dL, with two patients requiring hemodialysis prior to cardiac transplantation and Impella RP Flex implantation. All five patients experienced severe post-transplant cardiogenic shock (Society for Cardiovascular Angiography and Interventions [SCAI] classes D-E) prior to Impella RP Flex implantation and required further cardiac mechanical support.

### 3.2. Donor Characteristics

The mean total ischemic time for donor hearts in this cohort was 159.2 ± 67.2 min, with individual cases ranging from 89 to 238 min. Among the donor organs, three were preserved using cold static storage with the SherpaPak Cardiac Transport System (Paragonix Technologies, Waltham, MA, USA), while two were maintained with the TransMedics Organ Care System™ (TransMedics, Andover, MA, USA). All donor–recipient pairs demonstrated predicted heart mass ratios within acceptable transplant parameters, ranging from 0.85 to 1.2 (mean 1.006 ± 0.14).

### 3.3. Patient Outcomes

Following treatment with the Impella RP Flex, all patients demonstrated recovery of RV function. The mean duration of Impella RP Flex support was 8.6 ± 3.05 days (range 5–13 days, Table 2). One patient, who had undergone a combined heart and liver transplant, required subsequent implantation of a ProtekDuo (LivaNova, London, UK) MCS device 7–10 days after the Impella RP Flex’s removal due to persistent RV dysfunction and vasoplegia. Evidence of the uptake of biologic material was observed in one patient pump, but this was transient and did not affect the overall device performance. No cases of tricuspid valve injury were identified, and one patient experienced arrythmia during Impella RP Flex support.

### 3.4. Complications

The most common complications during RP Flex support were hemolysis, bleeding, and acute kidney dysfunction. Patient 1 experienced arterial bleeding from an arterial line placement unrelated to the Impella’s implantation and required brachial artery repair. Patient 2 required post-transplant transfusion due to persistent anemia. Patient 3 developed tamponade and hemothorax, necessitating intervention. Patient 4 underwent an open-chest procedure. Patient 5 experienced severe thrombocytopenia, resulting in excessive bleeding during the procedure. All patients experienced acute kidney dysfunction requiring hemodialysis. However, three of the patients recovered renal function prior to discharge, one regained kidney function within six months, and one, who was on dialysis upon admission, remained on dialysis at the time of this analysis.

## 4. Discussion

The results of this study indicate that the Impella RP Flex was a safe and effective modality for supporting a limited subset of heart transplant recipients who developed isolated RV-PGD in the immediate postoperative period and who did not require additional oxygenation support. Of the five heart transplant patients treated in this series, all survived to both 30 and 90 days post-transplant, and most (4/5) were ambulatory while still receiving Impella RP Flex support, underscoring one of the key advantages of the device: its small, percutaneous, and fully intracorporeal design that facilitates patient mobility and minimizes the need for external cannulation sites.

Although the cohort size here is small, the outcomes observed in this study are encouraging and consistent with prior data supporting the use of Impella RP in the management of acute RVF. In particular, our findings align with those of a published prospective study of 60 patients with RVF (including seven heart transplant patients) who were treated with the Impella RP device. The study reported a 73.3% overall survival rate to 30 days post-implant, discharge, or transition to the next therapy, suggesting that Impella RP support is both feasible and effective in the post-transplant setting [11]. Our study builds on that experience by focusing on use of the Impella RP Flex exclusively in a real-world transplant population.

The Impella RP Flex with SmartAssist is the only FDA-approved percutaneous RVAD indicated for use for up to 14 days in patients who develop acute right heart failure or decompensation within 48 h post-heart transplantation, provided that there is no profound shock, end organ failure, or acute neurological injury. Furthermore, according to the manufacturer (Abiomed), the Impella RP Flex with SmartAssist is contraindicated in patients with the following conditions: disorders of the pulmonary artery wall preventing the proper placement or positioning of the Impella RP devices; mechanical valves, severe valvular stenosis, or regurgitation of the tricuspid or pulmonary valve; thrombus in the right atrium, vena cava, RV, or pulmonary artery; anatomical conditions precluding pump insertion; and the presence of a vena cava filter or caval interruption device, unless clear access from the femoral vein to the right atrium exists to accommodate a 22 Fr catheter [12].

In this study, clinical selection for implantation of the device was based on a combination of physiological and anatomical criteria, including preserved left ventricular function, absence of blood oxygenation requirements, patient size compatibility, and patient-specific contraindications to VA-ECMO, such as difficult vascular or elevated bleeding risk. Importantly, the likelihood of patient survival was not a primary determining factor in the selection of the device.

Among the five patients in this cohort, there were no contraindications. The Impella RP Flex device was successfully utilized in two patients that were unable to receive anticoagulant therapy, including one patient that was undergoing a liver transplantation and experiencing severe thrombocytopenia and another patient that underwent an open-chest procedure and experienced subsequent bleeding.

Although all of the patients in our study exhibited hemodynamic instability and major derangements in the thromboelastogram, Impella RP Flex support proved to be safe and effective in patients experiencing RV-PGD. Adverse events observed in previously published clinical studies for the Impella RP Flex included major bleeding and hemolysis, which occurred in 48.3% and 21.7% of patients, respectively [11]. In our patient cohort, complications included bleeding unrelated to Impella placement and acute kidney dysfunction requiring hemodialysis. All patients recovered from these complications, except for one patient with preexisting renal compromise who remains on dialysis at the time of this analysis. Furthermore, there were no cases of tricuspid valve injury. Together, these observations suggest that with careful patient selection and therapeutic management, the Impella RP Flex can be used as a safe and effective treatment option for patients experiencing RV-PGD.

### Study Limitations

The limited sample size of this patient cohort significantly limits the ability to draw definitive conclusions regarding the efficacy and safety of the Impella RP Flex for the treatment of RV-PGD. As a retrospective case series, this study is inherently susceptible to selection bias and lacks a control group, which further constrains the interpretation of the findings. Clinical decision-making, including the precise timing of the device’s implantation and specific types and timings of adjunctive therapies, varied by patient, which may have introduced additional heterogeneity in the outcomes. Additionally, the absence of standardized criteria for RV-PGD severity further complicates interpretation. Lastly, the findings may not be generalizable to all transplant centers, given institutional differences in post-transplant management and device availability. Accordingly, larger prospective studies or registries are necessary to better characterize the optimal patient selection, timing of interventions, and long-term outcomes with the Impella RP Flex in this clinical setting.

## 5. Conclusions

The results of this study indicate that the Impella RP Flex device may represent a potential treatment option for post–heart transplant patients with isolated RV-PGD who do not require additional blood oxygenation, though further studies are needed to establish its safety and efficacy. While bleeding complications were common, they were manageable with standard interventions, and all patients recovered. The Impella device performed as intended, with no incidents of pump failure in this cohort. Its reduced catheter size (22–23 Fr for the RP Flex versus 19–46 Fr for VA-ECMO) [13] enables its use in small-stature patients for whom VA-ECMO insertion may be difficult or impossible. Additionally, the RP Flex allows patients the ability to ambulate during recovery. The primary complication observed in this study was acute kidney dysfunction, which resolved in most cases prior to discharge and was more likely attributed to the underlying RV-PGD rather than the catheter and hemolysis. In conclusion, given the pathophysiology, progression, treatment, and guidelines of RV-PGD, this study suggests that MCS support via the Impella RP Flex can be safe and effective when utilized in patients with isolated RV-PGD who do not require additional blood oxygenation and warrants further investigation.

## Figures and Tables

**Table 1:** Patient Characteristics.

	Pre-Impella RP Flex Treatment Patient Characteristics								Donor Organ Characteristics		
	Pre-Tx HF Etiology	PH	Inotrope * Dose (mcg/kg)	tMCS Device	Heparin IV Drip	SCr (mg/dL)	Type of Organ Tx	HD	Donor Death Classification	Ischemic Time (min)	PHM
**Patient 1**	NIDCM	Yes	0.25	IABP	Yes	0.9	H	No	DBD **	238	0.85
**Patient 2**	ICM	Yes	0.5	None	Yes	5	HK	Yes	DBD **	112	0.93
**Patient 3**	NIDCM	Yes	0.38	BiPella	Yes	1.3	H	Yes	DCD OCS	89	0.95
**Patient 4**	Tx CAD	No	None	None	No	1.3	H—redo	No	DBD **	223	1.2
**Patient 5**	NIDCM	No	None	None	No	0.9	HL	No	DCD OCS Vasoplegia	134	1.1

Patient treatment characteristics were obtained 24 h prior to initiation of the transplant surgery. Abbreviations: BiPella = biventricular Impella; DBD = donation after brain death; DCD = donation after circulatory death; OCS = Organ Care System™; H = heart; HD = hemodialysis; HF = heart failure; IABP = intra-ortic balloon pump; ICM = ischemic cardiomyopathy; IV = intravenous; K = kidney; L = liver; NIDCM = non-ischemic dilated cardiomyopathy; PH = pulmonary hypertension; PHM = predicted heart mass; SCr = serum creatinine; Tx CAD = transplant coronary artery disease; tMCS = temporary mechanical circulatory support; Tx = transplant. * Inotrope = milrinone. ** DBD hearts were cold-stored with a SherpaPak.

**Table 2:** Clinical parameters during Impella RP Flex support.

	Post-Tx DOS (Days)	P Level	Creatinine Pre-RP/Discharge (mg/dL)	Heparin IV Drip	Bleeding Requiring Transfusion	Hemodialysis
**Patient 1**	13	P7	2.6/1.2	Yes	Yes	Yes, Recovered
**Patient 2**	10	P6	4.8/1.8	Yes	Yes	Yes, Recovered
**Patient 3**	7	P6	0.6/4.6	Yes	Yes	Yes
**Patient 4**	8	P7	1.4/2.5	No	Yes	Yes, Recovered
**Patient 5**	5	P5	1.2/2.3	No	Yes	Yes

Abbreviations: Tx = transplant; DOS = duration of support; RP = Impella RP; IV = intravenous; P Level = Impella power level.

## Data Availability

Data will be made available upon reasonable request from the corresponding author.

## Abbreviations

The following abbreviations are used in this manuscript:

CI	Cardiac index
DOS	Days of support
ECMO	Extracorporeal membrane oxygenation
IABP	Intra-ortic balloon pump
MCS	Mechanical circulatory support
PCWP	Pulmonary capillary wedge pressure
RAP	Right atrial pressure
RV-PGD	Right ventricular primary graft dysfunction
RVAD	Right ventricular assist device
RVF	Right ventricular failure
SCAI	Society for Cardiovascular Angiography and Interventions
TEG	Thromboelastogram
tMCS	Temporary mechanical circulatory support
TPG	Transpulmonary pressure gradient
VA-ECMO	Venous arterial ECMO
VAD	Ventricular assist devices
